# Case report: Successful thromboprophylaxis with enoxaparin in a pregnant woman with internal jugular vein agenesis

**DOI:** 10.3389/fmed.2022.1011206

**Published:** 2022-11-22

**Authors:** Pierpaolo Di Micco, Luana Orlando, Donato Cataldo, Egidio Imbalzano

**Affiliations:** ^1^Unità Operativa Complessa Medicina, PO Rizzoli, ASL Napoli 2 Nord, Naples, Italy; ^2^Department of Clinical and Experimental Medicine, Polyclinic University of Messina, Messina, Italy; ^3^Unità Operativa Complessa Medicina, Frangipane Hospital, Ariano Irpino, Italy

**Keywords:** venous thromboembolism, internal jugular agenesis, thromboprophylaxis, enoxaparin, pregnancy

## Abstract

Internal jugular agenesis is a vascular malformation that is often associated with a history of recurrent headache. Due to the resulting abnormalities in intracranial venous drainage, it may be complicated by neurological dysfunction, such as intracranial hypertension, intracranial micro-thromboses, and neurodegenerative diseases such as multiple sclerosis. The simultaneous presence of jugular vein agenesis and thrombosis is possible in cases of acute illness, hormonal treatment, pregnancy, hypomobility, or venous drainage abnormalities (VDA) (e.g., May-Thurner syndrome). In particular, the literature still lacks data on thromboprophylaxis in pregnant women with jugular vein agenesis. Here, we report a positive experience with prophylaxis using enoxaparin during pregnancy in a patient with internal jugular agenesis.

## Introduction

The internal jugular vein is crucial for the drainage of intracranial veins (1). The literature contains numerous reports of venous drainage abnormalities (1–3), each of which is associated with the possibility of increased intracranial venous pressure and, thus, with relevant symptoms and signs such as headache, intracranial micro-thrombosis, intracranial hypertension, and seizures, as well as with neurodegenerative diseases (e.g., multiple sclerosis).

The presence of venous malformations, such as May-Thurner syndrome, internal jugular agenesis, or other types of vascular malformation associated with prothrombotic conditions such as pregnancy, may increase the risk of venous thrombosis. However, data concerning the association of venous malformation with venous thrombosis in pregnancy are lacking in the literature.

We may postulate that internal jugular vein agenesis may be associated with an increased risk of developing venous thromboembolism (VTE) in the context of additional prothrombotic risk factors such as pregnancy. An association between venous malformation and venous thrombosis in the presence of other prothrombotic conditions such as those reported above has, in fact, already been found in May-Thurner syndrome and vena cava agenesis (4, 5).

Here, we report an intriguing case of internal jugular vein agenesis in a pregnant young woman with a history of recurrent headache who underwent successful thromboprophylaxis treatment throughout her pregnancy.

## Case history

A 30-year-old pregnant woman was admitted to the outpatient clinic for thrombotic disorders at the Hospital Rizzoli in Lacco Ameno, Italy, due to an abnormal varicose vein located in her neck and a history of recurrent first-trimester miscarriage. During clinical evaluation, she specified that she was pregnant at week 8 (i.e., was experiencing pregnancy-induced amenorrhea). She was taking 200 mg of progesterone twice daily for miscarriage prevention (6), 15 mg calcium folinate, and 100 mg of aspirin because she was homozygous for MTHFR c677t (7). The patient's laboratory data are summarized in Table 1.

**Table 1:** Laboratory findings of reported patient.

**Test**	**Result**	**Normal values**
Factor V Leiden polymorphism	Wild type	Wild type
Prothrombin A20210G polymorphism	Wild type	Wild type
Protein S levels (%)	63%	50–120
Protein C levels (%)	65%	50–120
AT III levels (%)	71 (%)	60–120
d-dimer (ng/dL)	495	< 500
Fibrinogen (mg/dL)	396	200–400
Haematocrit (%)	31	< 45
Platelets (mmcube)	320,000	140,000–400,000
Homocysteine (mMol/L)	8	< 15

mg, milligrams; ng, nanograms; AT III, antithrombin III; LAC, lupus anticoagulant; antiβ2GPI, antibeta2 glycoprotein I antibodies; mm^3^, cubic millimeters; mMol, millimole. Anticardiolipin antibodies were dosed using fluoroenzyme immunoassay methods and kits, while antibeta2GP1 antibodies were tested *via* ELISA.

A differential diagnosis was made because of a positive history of recurrent miscarriage (Table 2) (8). Mild thrombophilia due to the predisposition to develop hyperhomocysteinemia (caused by the MTHFR 677 TT genotype) was the only detectable risk factor for recurrent miscarriage (defined as three spontaneous abortions during the first trimester of pregnancy without a detectable cause).

**Table 2:** Differential diagnosis performed by the patient for recurrent miscarriages.

Hysteroscopy for uterine malformation
Hormonal dosages to detect ovarian and thyroideal oligosynptomatic diseases
Uterine swab to detect uterine infections
Kariotype to look for chromosomal abnormalities
Immunological evaluation for antiphospholipid syndrome
Inherited thrombophilia

An elicitation of personal history to evaluate the presence of the unusual varicose vein did not reveal any previous surgical approaches, trauma to the neck, or the recent positioning of venous lines that could explain its development. Furthermore, no acute or chronic medical illness or acute infection was detected. For this reason, the probability of detecting an internal jugular vein thrombosis was considered low, and pharmacological antiplatelet treatment with 100 mg daily aspirin was confirmed.

The patient underwent a blood count and homocysteine check every month, as well as obstetric follow-up to prevent intrauterine growth retardation (IUGR).

**Figure 1:**
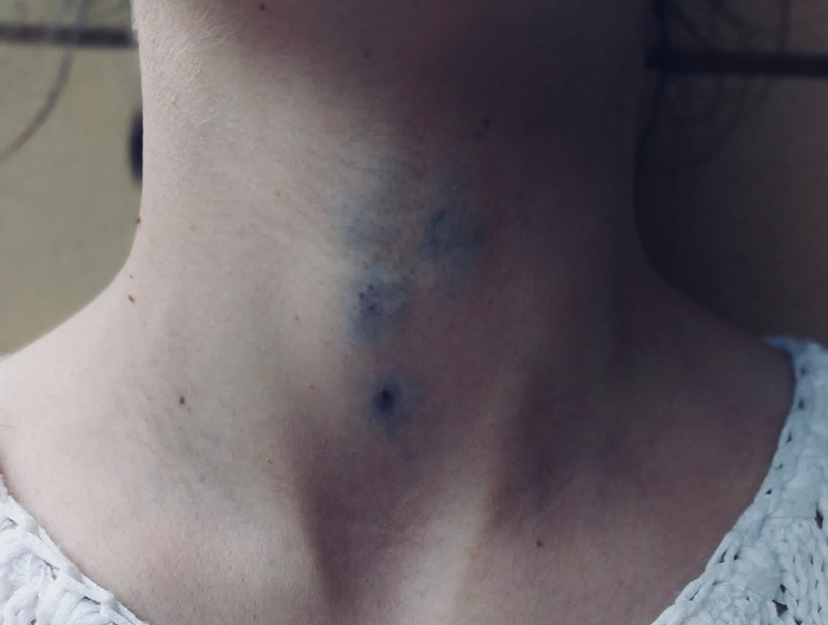
Collateral variceal venous circulation of the neck due to the presence of internal jugular agenesis.

However, 3 weeks after the first clinical evaluation, the patient was referred for an increase in the volume of the varicose vein in the neck, associated with cough (Figure 1). An color Doppler ultrasound examination was then scheduled and performed after a few hours.

**Figure 2:**
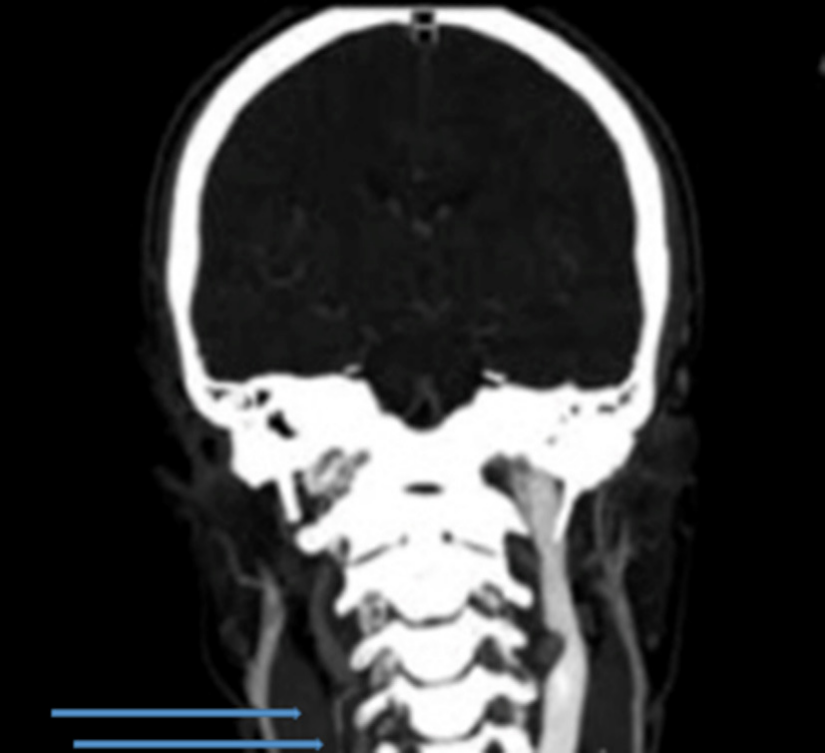
Magnetic resonance imaging with evidence of internal jugular agenesis.

The ultrasound scan revealed an enlarged left internal jugular vein with a large collateral venous circle involving the superior thyroidal vein, ipsilateral external jugular vein, and vertebral vein; on the right side, only the vertebral vein was visible, and it appeared to be of increased size. Therefore, the hypothesis of hypoplasia or agenesis of the right internal jugular vein was suggested. The blood flow in those veins was regular, and there was no ultrasonographic evidence of other vascular diseases. An MRI of the head and neck confirmed the hypothesis of venous agenesis (Figure 2).

Given the risk of jugular vein thrombosis, the use of a different method of thromboprophylaxis was considered. No guidelines are available in the literature, in fact, to suggest the proper approach in this clinical context, even though an increased thrombotic risk during pregnancy has been found for other venous malformations, such as May-Thurner syndrome and vena cava agenesis (4, 5). Our patient had several contemporaneous (if mild) thrombotic risk factors: a history of recurrent miscarriage, ongoing pregnancy and the associated hormonal treatment (although progesterone is less prothrombotic than other hormonal treatments), and venous malformation. For this reason, we stopped the administration of aspirin and we suggested starting a pharmacologic thromboprophylaxis with enoxaparin 40 mg daily until childbirth, to prevent the onset of venous thrombosis and also to prevent possible miscarriage.

Given the patient's atypical condition, periodic clinical and instrumental monitoring was planned, consisting of a monthly ultrasound of the veins of the leg and neck and an obstetric ultrasound to monitor uterine vascular pressure and gestational growth. Blood cell count, fibrinogen, prothrombin time, and activated partial thromboplastin time were also evaluated monthly (coagulation parameters and platelet count are often monitored during long-term prophylaxis with enoxaparin, particularly for pregnant women or patients with kidney failure, because of the modification of the half-life of low-molecular-weight heparin in these contexts).

No vascular complications were detected during the remainder of the pregnancy. The patient delivered a live-birth baby (weighing 3.0 kg) *via* cesarean section. Enoxaparin was prolonged for 4 weeks after delivery because of the surgical approach; the patient was then dismissed from our practice, having experienced no complications.

Written informed consent was obtained from the patient to describe her clinical picture.

## Discussion

Venous drainage abnormality is a silent condition that is not life-threatening for the patient. From a hemodynamic point of view, it is associated with low-flow and low-resistance veins, and it therefore confers a low risk of complications such as rupture or microthrombosis (9). With the increased use of MRI, microthromboses are more frequently being diagnosed (10, 11), as is reflected in the literature (12–14). However, when these abnormalities are associated with other prothrombotic conditions such as hormone intake, pregnancy, and acute medical illness, the potential risk for thrombotic complications in the cerebral vessels increases, as was recently demonstrated by the association between cerebral venous thrombosis and anti–SARS-CoV-2 vaccines (15, 16). At these sites, where the veins take a narrower and less linear course, conditions for the formation of venous thrombosis are more favorable. Nevertheless, after cerebral venous thrombosis, the hypothesis of an undiagnosed occult venous drainage abnormality (17) is frequently considered.

Prophylactic strategies to prevent venous thrombosis in the setting of venous malformation have been inconsistently explored in clinical practice and in the literature; prophylactic strategies to prevent thrombosis in cases of internal jugular vein hypoplasia or agenesis are even more limited. In our case, the venous drainage abnormality resulted from the absence of the right jugular vein, which hemodynamically favored increased inflow into the contralateral. This is even more interesting in the context of our patient, who possessed numerous mild risk factors for venous thromboembolism, such as pregnancy, obstetrical history of recurrent miscarriage, and hormone treatment. We therefore deduced an urgent need for thromboprophylaxis treatment, even though there were no precise guidelines or recommendations for a special case such as this one. We know, however, that pregnancy is a condition with a specific thromboembolic risk (18), and for this reason, there are special guidelines for the prevention, diagnosis, and treatment of venous thromboembolism in pregnancy (19, 20). Deep venous thrombosis (DVT) during pregnancy is associated with high mortality, morbidity, and health care costs (21). In particular, the last trimester of pregnancy seems to be the most dangerous in that it carries the highest risk of thrombosis, especially sinus thrombosis (22). During pregnancy, all three components of Virchow's triad are strongly represented: venous stasis, hypercoagulability, and endothelial dysfunction. Furthermore, increased uterine size results in slowed venous flow and blood stasis. This in turn also promotes endothelial damage. For this reason, after 30 weeks of gestation, there is a reduction in flow velocity, favoring the onset of deep venous thrombosis in the lower extremities and pelvis (23). Moreover, pregnancy-induced hypercoagulability, with its increased levels of coagulation factors II, VII, VIII, and X, as well as decreased levels of proteins S and C, contributes to thrombotic risk in addition to any pre-existing inherited predisposition to thrombosis (24).

Low-molecular-weight heparin is most commonly recommended for the prevention of venous thromboembolism in pregnancy (19). In contrast, there is no indication for direct oral anticoagulant therapy (20) during the gestational months, while low-dose aspirin prophylaxis (81 mg/day) is recommended only for women at high risk of pre-eclampsia and should be initiated between 12 and 28 weeks of gestation (25).

## Conclusions

We reported a singular case of a pregnant woman with a rare vascular malformation, mild thrombophilia, and a risk of developing venous thrombosis. This anatomical venous malformation is only rarely described in case reports, and has not yet featured in randomized clinical trials. Because the patient was at risk of developing thrombotic complications, such as miscarriage or venous thrombosis, due to her medical history, we performed a thorough clinical evaluation of all clinical conditions and risk factors that may favor the adoption of a prolonged pharmacological thromboprophylaxis. As we await larger clinical trials to confirm the validity of this method, we hope that our precautional medical approach can be of use in similar cases.

## Data availability statement

The raw data supporting the conclusions of this article will be made available by the authors, without undue reservation.

## Ethics statement

Ethical review and approval was not required for the study on human participants in accordance with the local legislation and institutional requirements. The patients/participants provided their written informed consent to participate in this study.

## Author contributions

PD wrote the draft. EI reviewed the draft. DC and LO took clinical information. All authors contributed to the article and approved the submitted version.

## Conflict of interest

The authors declare that the research was conducted in the absence of any commercial or financial relationships that could be construed as a potential conflict of interest.

## Publisher's note

All claims expressed in this article are solely those of the authors and do not necessarily represent those of their affiliated organizations, or those of the publisher, the editors and the reviewers. Any product that may be evaluated in this article, or claim that may be made by its manufacturer, is not guaranteed or endorsed by the publisher.
